# The Role of Trust in the Care of Young Children with Type 1 Diabetes

**DOI:** 10.3390/children8050383

**Published:** 2021-05-12

**Authors:** Patricia DeCosta, Timothy Charles Skinner, Dan Grabowski

**Affiliations:** 1 Diabetes Management Research, Steno Diabetes Center Copenhagen, 2820 Gentofte, Denmark; dan.grabowski@regionh.dk; 2 Department of Psychology, University of Copenhagen, 1353 Copenhagen, Denmark; ts@psy.ku.dk; 3 Department of Rural Health, La Trobe University, Bendigo, VIC 3552, Australia

**Keywords:** trust, young children, type 1 diabetes, psychosocial, child-centred care

## Abstract

Using the theoretical framework of Guido Möllering conceptualising trust as a mental process composed of three elements—expectation, interpretation and suspension—we examined the role of trust in relation to young children’s (age ≤ 7 years) psychosocial needs when diagnosed with type 1 diabetes. Based on qualitative interviews with health care professionals (HPCs) from paediatric diabetes clinics in all regions of Denmark, we identified four main themes: trust through meaningful interaction, trust as a key factor at the time of diagnosis, trust in a long-term perspective and caregivers as the bridge to trust. We conclude that trust between young children and HCPs is central to children’s psychosocial experience, as well as a primary need, when children are diagnosed with type 1 diabetes. Trusting relationships counteract children’s experience of fear, anxiety and needle phobia and reinforce HCPs’ experience of providing good psychosocial as well as medical care. The present study offers insights into how trust can positively affect young children’s experience of diagnosis. This study also points out some key barriers to and facilitators of creating trusting relationships. This research is a first step towards a greater understanding that can inform collective future guidelines on the psychosocial care of young children.

## 1. Introduction

Type 1 diabetes is a chronic illness requiring daily treatment, which involves daily monitoring of blood glucose concentration and subcutaneous injection of insulin. The number of children diagnosed with type 1 diabetes has been steadily increasing [1], with the number under the age of 5 increasing most rapidly [2]. In addition to the medical implications of living with type 1 diabetes, there are well-established psychosocial consequences of living with this chronic illness that affect the entire family [3,4,5]. Research has consistently found a greater incidence of psychological distress among children and young people with type 1 diabetes compared to their peers [6].

While the importance of psychosocial factors in the care and management of type 1 diabetes in childhood is recognised [6], guidelines from the American Diabetes Association on psychosocial care do not currently address children under the age of 7 years [7]. The absence of guidelines reflect a lack of research and knowledge concerning this age group [8] and a tendency for the views of younger children to be excluded at the clinic level [9]. Across age groups, the available literature mainly explores the experiences, rather than the needs, of children diagnosed with type 1 diabetes. Thus, the authors of a recent review found it challenging to identify the need for support, knowledge that could inform the development of interventions for these children [10].

Identifying and understanding young children’s experiences, as well as their need for support, are the foundation and prerequisite of offering child-centred care, as well as of developing meaningful psychosocial interventions for children. Thus, the purpose of the present study was to gain insights into young children’s experiences and needs when they are diagnosed with type 1 diabetes, and to do so through the health care professionals (HCPs) caring for these children. In the present study, the term ‘young children’ refers to children 7 years of age and below.

### 1.1. The Role of Trust

From early on, trust has been a central concept in developmental psychology, and trust research has largely been shaped by either Erikson’s or Rotter’s theoretical position [11]. According to Erikson, trust is established from birth in response to reliable and consistent care from the primary caregiver [12], whereas Rotter views trust as a predominantly learned cognitive construct [13]. In the case of preschool children, trust has traditionally been examined (albeit indirectly) in the context of attachment theory [14], pioneered by Bowlby and Ainsworth [15]. However, while few psychological studies have examined peer trust in relation to promise- and secret-keeping behaviour in young children [16], an understanding of the role of trust in the context of adult authority and social institutions is lacking for this age group [11].

In adults, the research indicates that trust plays a key role in the health care setting. Patient trust in HCPs is positively correlated with outcomes, such as adherence to treatment recommendations, perceived effectiveness of care and improvement in self-reported health [17]. In adults, willingness to trust in health care providers has not been found to be a psychological trait. Rather, the literature suggests that most patients will enter such encounters with the potential to trust [17]. However, to our knowledge, the current literature does not address the role of trust, in health care, in this young age group.

### 1.2. Social Theory of Trust

While developmental theories of trust offer a framework for understanding how young children’s ability to trust is shaped by their experience and interaction with a primary caregiver, in regard to our data, these theories have less to offer in the form of a framework for understanding the role of interpersonal trust between young children and their HCPs. Hence, we look to social theories of trust for a framework to guide the analysis of our findings.

Sociology continues to be central to understanding the role and complexity of trust in the health care system [18]. From a sociological perspective, trust is a multifaceted social experience encompassing cognitive, emotional and behavioural dimensions. Further, trust is studied as a quality shared between people, rather than as a psychological state of isolated individuals [19]. In essence, trust can be defined as a state of favourable expectations concerning other people’s intentions and actions [20].

Relevant to the present context of the health care setting, key trust theories recognise two forms of trust: institutional and interpersonal [18,19]. Simmel argued that the institution associated with and the position of an individual have become so reliable that trust now rests, to a lesser extent than previously, on personal knowledge and qualities [21]. Likewise, Luhmann argued that when lack of time and familiarity render opportunities for interpersonal trust non-existent, people rely on their generalised trust in the medical system, thereby enabling trust in a representative of this system [22]. In contrast, Giddens argued that interpersonal trust is a prerequisite of trust in the (health care) institution [18].

The interplay between institutional and interpersonal trust is pertinent to the focus of our research, as young children’s knowledge and understanding of the health care system is bound to be limited. This leads to an important question regarding what ‘anchors’ of safety young children can rely on if they cannot draw on generalised trust in the health care system and do not have the ability to rationalise the need for medical intervention. Indeed the major social theories on trust has to be criticised for not adequately recognising the role of social factors, such as age [18].

Thus, the primary aim of the present paper is to understand the role of trust in relation to young children’s psychosocial needs when diagnosed with type 1 diabetes. Secondly, we aim to study the trust-building process between young children and HCPs, the goal being to elucidate key bases for and barriers to trust.

### 1.3. Theoretical Framework

#### 1.3.1. The Simmelian Notion of Trust—A Simple Model

*“Trust can be imagined as the mental process of leaping—enabled by suspension—across the gorge of the unknowable from the land of interpretation into the land of expectation”*

[20]

To conceptualise and define trust and to frame our analysis, we draw on the theoretical framework of Guido Möllering [20], which is based on the work of Georg Simmel [21,23]. The Simmelian notion of trust is a model that conceptualises trust as a mental process consisting of three elements: expectation, interpretation and suspension. Möllering [20] identify Simmel’s influence on the conceptualisation of trust and his continued influence on trust research chiefly through the work of Luhmann [22], Lewis and Weigert [19], as well as Giddens [24,25]. Though the above-mentioned writings have greatly influenced the conceptualisation of trust, we do not draw directly on these in the present analysis.

#### 1.3.2. Expectation, Interpretation and Suspension

Expectation describes the state reached at the end of the trust process, which may be positive or negative. In this way, a positive expectation concerning another person’s intentions is the output of the trust process [20]. According to Simmel [21] and Möllering [20], the bases of trust and the state of expectation are only weakly connected. Therefore, it is important to differentiate between expectation and the action positive expectation may lead to—for example, cooperation. Cooperation is not always the result of favourable expectations (e.g., power or dependency), and a favourable expectation may not always result in cooperation. Interpretation is the element through which individuals’ subjective experiences of their lifeworld form the bases of or ‘good reasons’ for trust. Möllering explains that, according to the Simmelian notion of trust, there is a gorge between expectation and interpretation. This gorge represents the unknown. Interpretation represents the bases of trust and serves as the platform upon which the leap of trust (suspension) is made [19,20]. Finally, the third element of suspension—as conceptualised by Möllering—is based on Simmel’s notion of trust as a mental leap of faith, while also being inspired by Luhmann and Giddens [20]. Giddens [25] describe the leap as momentarily bracketing ignorance or lack of knowledge. Through suspension, uncertainty and ignorance are momentarily suspended, rendering the uncertain momentarily certain and enabling the leap from interpretation (good reasons or bases of trust) to the state of expectation [20,25]. According to Möllering, suspension, while it cannot stand alone, is the defining element of trust.

Thus, the process of trust can be said to start with interpretation, where ‘good reasons’ for or the bases of trust are shaped by people’s lived experiences and end with the state of expectation. The state of expectation can be favourable, resulting in a state of trust, or unfavourable, resulting in a state of distrust. Connecting the two is the final and defining element of trust, suspension, which enables the leap of trust from interpretation to expectation. However, note that the trust process is non-linear; when the state of expectation is reached (favourable or unfavourable), this experience will again feed into interpretation. In this way, by studying and connecting all three elements, the model can be used to understand how people trust [20].

Applying the theoretical framework to the data will help us to consciously and critically differentiate the concept of trust from related concepts. The framework also calls for moving towards a hermeneutic approach, thus avoiding reducing research on trust to its functional properties [20]. This is important to be mindful of in our quest to answer the research questions: What is the role of trust (output of the trust process)? How is trust encouraged (input/bases for trust)? Further, as previously discussed, the major social theories of trust have been criticised for not adequately recognising the role of social factors, such as age [18]. We suggest that Möllering’s framework of trust acknowledges the complex, multidimensionality of trusting relationships and can help to explain the role and conditions of trust from the perspective of young children in the context of diabetes care, from diagnosis and beyond.

## 2. Materials and Methods

All 17 paediatric diabetes clinics in Denmark were invited to participate in this qualitative, interview-based study, which took place between December 2018 and January 2020. Twenty health care professionals (HCPs) from 11 clinics, representing all five regions of the country, accepted the invitation. This included 11 paediatric nurses, four paediatricians, three psychologists and two dietitians.

Individual interviews were conducted face to face at the HCPs’ respective home clinic. The interviews were semi-structured, permitting an open conversation about young children’s psychosocial needs when they are diagnosed with type 1 diabetes. The interview guide consisted of 18 open-ended questions regarding young children’s psychosocial needs (e.g., What do you believe is the primary need of young children, when diagnosed with diabetes and during the period afterwards?); their experiences (e.g., How do you gain insight into children’s experiences and emotions?); their challenges (e.g., What does it look like when a child is struggling with a diabetes diagnosis); and their strengths (e.g., What do you believe children’s greatest strengths and competences are in regard to handling diabetes?). The interview guide did not include any direct questions about trust. Over a period of two weeks, prior to data collection, the first author (PD) passively observed a number of paediatric diabetes consultations at a clinic in Denmark. The observations were not used in the analysis, but provided a contextual background for the interviews. The interviews lasted between 23 and 50 min. PD was responsible for conducting all of the interviews. PD and a student assistant transcribed all of the interview data verbatim.

### Analysis

The interview data were analysed using thematic analysis in accordance with Braun and Clarke [26]. In Phase 1, preliminary analysis of the data was carried out by listening, reading, rereading, and getting familiar with the data. Secondly, the transcribed dataset was transferred to the qualitative data analysis program Nvivo for detailed coding (Phase 2). In Phase 3, we used the theoretical framework conceptualised by Guido Möllering [20], based on the work of Georg Simmel [21,23], to guide the data analysis. In this way, trust in relation to the research questions evolved through the initial coding process in Phases 1 and 2. The prominence of trust as an overarching theme was revealed through inductive analysis. However, further analysis and subsequent formation of themes were driven by theory, resulting in a more detailed analysis of the data related to trust.

In Phase 3, we identified all of the codes related to trust, as defined by the theoretical framework, as we scanned the remaining dataset for overlooked data related to trust. Secondly, we refocused our analysis to the broader level of themes by constructing and refining a thematic map [26]. In addition to reviewing the themes in relation to coherence in Phase 4, we also explored how each theme and the corresponding data sections related to the three elements of trust: expectation, interpretation and suspension. Concurrently, the framework was applied to the data, allowing us to explain our findings in relation to trust as a dynamic process. In this way, the theory enabled us to identify even subtle elements of the trust-building process through the HCPs’ narratives. During each phase, all authors (PD), (TS) and (DG) took part in the discussion, and consensus was reached regarding the final themes.

## 3. Results and Discussion

We identified four main themes in the analysis: trust through meaningful interaction, trust as a key factor at the time of diagnosis, trust in a long-term perspective and caregivers as the bridge to trust. Although distinct, the themes intrinsically link to the meaning of trust in the psychosocial care of young children with type 1 diabetes.

### 3.1. Trust through Meaningful Interaction

Trust was mainly described by HCPs in relation to forming trusting relationships. Trusting relationships were created through interaction, which was meaningful for the children. Building trusting relationships with young children and their caregivers was described as indispensable, or as a prerequisite for providing good diabetes care.

*‘Well, I think it’s absolutely essential to have a connection. And I think; if I don’t have that relationship, then I don’t think I can help them the same way—because I don’t think I will be heard the same way. So I have to have a connection and some sense that this, –this is not something dangerous.’*

—Paediatrician (19)

*‘After all, I think it is the primary thing; to develop a good connection with those children. If you don’t establish a good relationship with the family and children, you won’t get anywhere. This is the place to start.’*

—Paediatric nurse (17)

The HCPs likened the absence of a connection with a child as the absence of trust, which in turn resulted in a sense of being ‘stuck’ and a barrier to effective communication. This indicates that the relationship between the HCP and the young child is a central factor allowing (or preventing) the child to move forward—enabling a leap of trust. In this way, the relationship itself represents suspension. Consistent with the theory, interpretation and suspension always combine. That is, the leap of trust cannot be made from nowhere—it is made from the place of interpretation. Accordingly, every meaningful interaction between the child and the HCP fed into interpretation and good reasons for trust.

According to the HCPs, trusting relationships were formed through meaningful interaction and had little to do with the HCPs’ medical abilities or diabetes knowledge. On the contrary, the HCPs talked about the value of forming a connection with the child, where diabetes was not part of the interaction. Conversations about diabetes were, for the most part, not meaningful to the young children.

*‘[…] I believe that the child should know me as someone other than the lady who comes and only talks about things to do with diabetes. So that may well be a walk where we don’t talk about it at all, but where we just go for a walk. […] I think it can add a lot of value to the relationship, in that the child also gets to know me a little bit and finds out who I am and that we can have some fun. For the most part, these pre-schoolers, they want to play a lot and have a lot of fun. And if you can represent that, then you can also successfully represent something that is perhaps not so much fun and not so entertaining.’*

—Paediatric nurse (13)

This quote captures the trust-building process and its unique value and role in paediatric care for young children. If the HCP is willing to step out of his/her role as a diabetes nurse and into the world of the child, the child will correspondingly be more willing to accept the necessary medical treatment. Meaningful interaction entails creating space (physically and mentally) and entering the lifeworld of the child. One HCP called this ‘speaking the language of the child’—Paediatric nurse (16). The lifeworld of young children was described as a world where communication was accomplished through play, body language, humour, imagination, storytelling and singing.

*‘Now I think we have even more focus on the fact that we need to reach them through play –whenever possible. So you have to, of course, you have to invest a bit of yourself and you have to make a fool of yourself once in a while. Once I was lying, together with the [hospital] clown (Hospital Clowns (Danske Hospitalsklovne) is a privately founded charity that supports hospitalised children and their families [27]), on the floor in our reception room with little Peter, when he was brand new [newly diagnosed]. […] I sang the Andrea song [Danish children’s song] while she distracted him and I could prick him without him noticing. So sometimes, you have to get out of your comfort zone, that is –dare to be a little silly. But I also think it’s part of what makes the kids think; that it’s OK after all! […] It offers some kind of reassurance when you dare to sing like Andrea.’*

—Paediatric nurse (1)

This indicates that the HCPs’ willingness to enter the child’s lifeworld is meaningful to young children, reassuring them that they are in safe hands. In this way, meaningful interaction feeds into to the child’s interpretation to form good reasons for trust. The HCP quoted above described entering the child’s lifeworld as ‘risking making a fool out of yourself’ or ‘to invest some of yourself’. This implies that the HCP is also taking a risk—or a leap of faith—by putting his/her trust in the child. ‘Getting out of your comfort zone’ and into the child’s lifeworld was evidently reciprocated by children’s increased willingness to co-operate and accept medical treatment. Thus, the exchange of trust could be interpreted as a functional outcome of a (favourable) expectation. In this way, the HCPs’ willingness to enter young children’s lifeworld was evidently interpreted as an expression of trust, creating and reinforcing a positive cycle of interpretation (entering the child’s life-world/taking a risk), suspension (the relationship itself), leading to a favourable expectation (trust).

At first glance, distraction may have more to do with directing the child’s attention away from fear and anxiety, rather than towards trust. With distraction, the child does not have to make the leap. The procedure is done without explicit consent. However, in this way, the medical procedure is done without restraint, fear and ensuing negative expectation. Thus, reducing fear and anxiety may alter the child’s experience of pain, feeding positively into interpretation, thus creating a strong foundation for and positive continuation of the trust process.

Importantly, when asked about signs indicating that a child is struggling with aspects of the diagnosis, the HCPs mentioned the inability to establish a connection with a child.

*‘Their Body Language when They Come in, Quiet Children that You Have Poor Connection with. It Can Even Be Very Young Children.’*

—Paediatric nurse (17)

### 3.2. Trust as a Key Factor at the Time of Diagnosis

The acuteness and intensity of diagnosis were described as barriers to establishing trusting relationships. The combination of pain, an unfamiliar environment and strangers created a difficult starting point for cultivating trust. Young children’s lack of understanding, coupled with the need for urgent medical intervention, made diagnosis especially stressful for young children.

*‘In the space of just a few hours, there will be a doctor, sometimes two, and a nurse and a phlebotomist that pricks them, and then a whole lot happens. […] if you only see it from the child’s perspective, then it must be a disaster, because they don’t understand what’s happening.’*

—Paediatrician (10)

In particular, the experience of pain associated with the diagnosis was a major barrier to HCPs’ ability to establish trusting relationships. The HCPs described how young children’s experience of pain shaped their understanding and future expectations of diabetes treatment. They emphasised the impression that pain made on children in this age group and how ‘starting off right’ was important to reducing the risk of excessive fear and needle anxiety.

*‘There’s no doubt that an experience of pain is bad. But it’s not always as simple as that, because the pain can start long before a needle is even in sight. So children’s pain is very important […] and then of course you have to have a lot of ‘magic cream’ –you should not insert a needle into children who are not locally anesthetized. Oh, and if you can’t manage to take the blood tests, then you should not attempt to do it in the other arm, unless local anaesthetic cream has been applied there as well. Because we are remembered for that –very much so.’*

—Paediatrician (19)

The above indicates that young children’s experience of pain creates a strong and unfavourable expectation (distrust) of the HCP’s intentions. As the process of trust continues, the negative expectation may feed into and shape interpretation. Without adequate opposing trust-building experiences, this may lead to a further negative spiral of distrust. Accordingly, the HCPs explain how extreme fear experienced at diagnosis may lead to a downward spiral of restraint, pain and lack of positive expectation (trust).

*‘The pre-schoolers, who I believe are not doing that well, they react with resistance. Already when I open the door to the room they begin crying and really show resistance to; oh no, not the woman in the white clothes again! They are extremely difficult to calm down. Extremely difficult even to get near. And it’s a bit of a downward spiral, because then I have to force myself on them. And sometimes I have the opportunity to pull away and say; okay then we’ll look at something later—and sometimes I have the opportunity to try to approach them by playing with cars on the floor or blowing bubbles or something like that. But if the whole situation is so tense that the whole family is affected, greatly affected by this, then it can be difficult anyway. And then there is crying and resistance.’*

—Paediatric nurse (1)

The bases of trust and the state of expectation are only weakly connected. Thus, the experience of pain does not necessarily lead to distrust and lack of cooperation on the part of the child. Trust is a dynamic process, where many factors influence how children experience and interpret their lifeworld. This was reflected in our data, where the HCPs were able to establish trusting relationships despite the difficult circumstances surrounding diagnosis. However, this was an active process in which the HCPs engaged in reinforcing positive bases of trust, such as entering the child’s lifeworld and engaging in meaningful interaction.

Hence, a strong precondition for building trust at diagnosis was being patient and respectful in the encounter with the children. The HCPs spoke about taking things slowly and being respectful of the child’s initial apprehension and hesitation.

*‘Yes, joke with them, show respect, don’t crowd them, that is, instead approach them little by little and let them decide the speed. I believe so. But I don’t know, you probably can’t have a deeper conversation with them, but you can do a few simple things with them. Maybe use some toys too.’*

—Paediatrician (18)

Trust involves a degree of familiarity, as familiarity is needed to inform interpretation or ‘good reasons’ for trust. Thus, time is a prerequisite of the process of trust taking place. However, as reflected in our data, familiarity itself does not lead to either favourable or unfavourable expectations, instead it allows the potential for both states to be achieved. One HCP explained the impact of neglecting the relational work and going straight to medical procedures when dealing with young children. She told a story of being called urgently to the hospital to initiate diabetes treatment to a young child, before rushing off home to get to a personal appointment:

*‘Because of this, for over half a year, whenever Hannah saw me, she said “Ow”. Moving past it has taken a really, really long time. Because I just came out of nowhere, stuck a needle in her and left again. And it just confirms to me that this is the wrong way to do it. It’s so important that you take your time getting properly familiar with these children.’*

—Paediatric nurse (1)

Consistently, our data revealed that allowing adequate time—from hospital admittance to medical intervention—was critical to young children’s experience. Time allowed the HCPs to engage in the process of trust formation, permitting a certain familiarity to develop as well as allowing pain management procedures to take place. As illustrated in the quote above, attending to the child’s medical needs only, and neglecting psychological needs, leads to distrust. As a result, the HCP was faced with the clearly more challenging process of repairing trust.

Following diagnosis, familiarity continued to play an important role in enabling young children to predict the likelihood of anything unforeseen, possibly unpleasant, occurring. All of the HCPs emphasised the importance of young children consistently seeing the same nurse and doctor for consultations at the clinic.

*‘She came here once and there was a doctor (in the room), she immediately held on tight to her father. So the doctor said; what’s going on here? The father said; it’s because there’s someone new here, someone she doesn’t know—then she doesn’t know what’s going to happen.’*

—Paediatric nurse (14)

As evident in the data, familiarity and predictability were essential to making the young children feel safe. Meeting someone familiar in the clinic was part of how children made sense of the situation, and it enabled them to predict what was going to happen during the consultation. If the child did not recognise the HCP or the association was only that of pain and fear, the child was wary, unable to trust in the good intentions of the HCP.

### 3.3. Trust in a Long-Term Perspective

Establishing trusting relationships with young children was discussed as a long-term ‘relational investment’. The HCPs recognised that diagnosis was the start of a relationship that in most cases would continue into young adulthood. A relationship continuously built on trust would allow teenagers and young adults to disclose behaviours that critically affect their diabetes management, such as drinking alcohol or taking recreational drugs.

*‘It starts as soon as they step through the door. Because if I, when they’re little already, don’t prioritise getting to know these children and don’t spend time playing with them and create a sense of safety and those kinds of things. Well then, they will never be able to trust me and then they’ll never tell me anything. So the foundation of the relationship starts on day one.’*

—Paediatric nurse (1)

The HCPs described trust in relation to a process of building trust during the early years, continuing to foster trust throughout childhood, thus indirectly affecting their ability to provide good diabetes care as these children grow older. This reveals that trust is a continual process, where expectation, favourable or unfavourable, becomes interpretation, which in turn enables (or hinders) suspension. Each input and interaction, (especially) the early ones, inform and shape the process of trust by feeding into and connecting each of the three elements.

*‘But you already start to build trust and some kind of confidentiality at that time, in order to –Because, when she turns fifteen and I have to have a consultation with her, without her parents, and then all of a sudden we’re going to talk about birth control pills and alcohol, right? So it starts early. So I think working with the little ones is not so much about talking about diabetes, it’s about establishing so they know […] a relationship. So they know what it’s all about, right? […] because if you take a consultation, for example, where the child is lying on the bed and you talk right over the kid with the parents. Well, then you’ve already—right there, you’re losing that game.’*

—Paediatrician (2)

### 3.4. Caregivers as the Bridge to Trust

The HCPs described how working with young children entailed simultaneously cultivating a trusting relationship with the children and with their caregivers.

*‘It is the parents you give all the information to, but at the same time you must also make the child feel safe. Because I have to do a lot of things to little Sophie, so she has to feel safe with me too. But at the same time, I have to make mum feel safe, by giving her as much information as possible. So there are two sides to it: On one side, there is the relationship formation with Sophie, which must include play, but sometimes I also have to do things that she thinks are a really bad idea. Like inserting an Iport, putting on an insulin set or measuring blood sugar in her finger. And then at the same time I have to make mum and dad feel safe.’*

—Paediatric nurse (1)

The HCPs explained how the parallel process of building trust with young children and caregivers had a dual purpose. Naturally, being diagnosed with diabetes affects the whole family, and the HCPs have to train caregivers to carry out diabetes management for their children. However, a trusting relationship between the caregiver and the HCP evidently also enhanced the child’s perception of safety, enabling the child to indirectly put his or her trust in the HCP.

*‘Yes, so they really mirror us through their parents. That is, if the parents are comfortable with what we do and what we say, then it’s also very clear that the child—it can be slightly different when you have a teenager, they may well ask some more questions themselves. But those smaller children, they really mirror their parents’ reactions to us, as health professionals.’*

—Paediatric nurse (13)

*‘The thing about cultivating the connection with the parents, also in order to make them (the child) feel that it’s okay: We have a good collaboration here –and then he feels safe being here.’*

—Paediatric nurse (3)

It was evident that the young children were able to, in a sense, ‘bracket’ their own lack of understanding—or even lack of trust—instead relying on the trust that their caregiver expressed in the HCPs. This enabled the children to accept medical treatment in a reasonably calm matter.

*‘While those who do well may be crying too, but there’s a difference between crying and struggling with flailing arms and legs. Then there are some (children) who sit quietly and cuddle up to their mother and cry. Clearly showing that; I think this is a bad idea, but find comfort by nestling into mum or dad. […] None of them think it’s a great idea that I fit the pump on their stomach or measure blood sugar in their finger. But some just manage to find the strength to say; if I cuddle up really close to my mum or dad, then you can do what you need to do, out there –and then it’s over.’*

—Paediatric nurse (1)

In this instance, behavioural compliance is not necessarily a consequence of the child trusting the HCP, but rather a result of trusting the caregiver. The child relies on interpretation and favourable expectations in relation to the caregiver, in order to make a leap of trust. In this way, the caregiver may bridge the HCP’s initial lack of time, familiarity and opportunity to build trust with the child.

## 4. Discussion of Theory and Main Conclusions

Möllering’s theoretical framework, which conceptualises trust as a mental process, proved valuable and appropriate to our aim of understanding the role of trust in relation to young children’s psychosocial needs when they are diagnosed with type 1 diabetes. Further, the framework was useful in identifying key bases of, as well as barriers to, trust, while acknowledging the complex, multidimensionality of interpersonal trust.

Not surprisingly, given the cognitive maturity of young children, we found no indication of generalised trust in the health care system or HCPs in our data. On the contrary, a white coat—a symbol of the health care system—had the opposite effect on some children, resulting in wariness and fearfulness. Although this aspect was not thoroughly covered in our data, a child-friendly hospital environment may play some role in generalised trust, through recognition of a non-threatening and familiar environment [28], because system trust is activated by the sense of ‘everything seeming to be in proper order’ [22]. In our data, the children clearly relied on their trust in their parents to reduce complexity, bracket uncertainty and make the leap of trust—despite lack of time, familiarity and opportunity to build trust. This extension of trust through trust in parents could be interpreted as the occurrence of a form of generalised trust.

While our data suggested that young children relied on the safety offered by their parents in order to bridge their lack of trust in the HCPs, based on our findings, we cannot speculate as to how early attachment and experiences of trust between children and their caregivers affect children’s ability to trust HCPs. In our data, the HCPs described instances in which the young children’s resistance and fear had made it very difficult to find opportunities for building a relationship, and thus trust. Clearly, some children lacked the ability, or the fundamental sense of safety, to make even the smallest leap of trust. In these instances, lack of secure attachment, responsive caregiving and the reliability of a caregiver may play a role.

As evident in our analysis, trust formation was central to young children’s experience of pain, fear and needle anxiety. Research has demonstrated that young children trust other people who do nice things, rather than people who merely say nice things [29]. It is clear that, in this age group, actions speak louder than words. Further, research has shown that the experience of pain is significant to children in this age group. Needle anxiety is far more prevalent among younger (<9 years) compared to older children [30,31] with type 1 diabetes. As reflected in our data, this highlights why the association between pain and the HCP is a significant obstacle to the formation of trust. In Möllering’s framework [20], there is no automatic logic connecting interpretation with expectation. Favourable and unfavourable reasons may coexist. This was apparent in our data, where cooperation occurred without established trust (e.g., trust mediated through parents) and the process of trust could occur despite the experience of pain. By engaging in other bases of trust, such as allowing time for familiarity and meaningful interaction to occur, the HCPs managed to enable children to continuously make the leap (suspension) of trust across that gorge of the unknown.

Further, from a life course perspective, trusting in a HCP’s good intentions may alter the child’s experience of ‘violation’ at the time of diagnosis—even in a situation where a child is not wilfully cooperating. In other words, if a long-term trusting relationship is the goal, fear and pain cannot be the only experiences informing interpretation. In accordance with social theories of trust, familiarity itself does not lead to trust. In adults, trust is only weakly associated with the length of the doctor-patient relationship, indicating that trust is mainly established early in the relationship [17]. Using Möllering’s framing of trust as a continual mental process, we can better understand our findings, which are that trust built early in the relationship, even in the case of young children, continues to affect trust in the years that follow.

In our data, the HCPs’ willingness to step out of their professional roles and into the lifeworld of the children also seemed particularly valuable in facilitating trust. Viewing trust through the lens of social theories allowed us to elucidate one possible mechanism facilitating this trust: In addition to engaging in a way that was meaningful to the children, the HCPs also displayed trust in children by ‘putting themselves out there’, implying risk-taking on the part of the HCPs. This is in line with Luhmann’s notion that when others act in a way that implies trust in us, we tend to reciprocate by trusting them [22]. Or as Simmel put it: “the trust we receive contains an almost compulsory power, and to betray it requires thoroughly positive meanness” [21]. In this way, the act (willingness to enter the lifeworld of the child), more than the specific activity (singing, playing etc.), facilitates the formation of interpersonal trust and the bases of trust.

Concerning the aim of the present study, we conclude that trust between young children and their HCPs is central to young children’s psychosocial experience, as well as a primary need, when children are diagnosed with type 1 diabetes. Through the formation of trusting relationships, the experience of fear, anxiety and needle phobia is counteracted. Further, efforts invested in the trust-building process at diagnosis positively affect future interaction that requires older children to disclose sensitive information about themselves that may affect diabetes management. Trust enhances the HCPs’ experience of providing good psychosocial and medical care. Time, familiarity and predictability increase young children’s ability to trust. Finally, trust between young children and HCPs is encouraged when the HCPs step out of their ‘medical’ role and engage in activities that are meaningful to young children.

### 4.1. Implication for Practice and Future Research

Our results suggest that the process of building trusting relationships should be actively imbedded in diabetes care for young children. Focusing on the trust-building process may be particularly pertinent at diagnosis, when young children are in need of non-verbal, non-rational safety cues. The present study hopes to provide HCPs with an understanding of *how* trust can positively affect young children’s experience of a diagnosis. This study also points out some key barriers to and facilitators of creating trusting relationships.

Our results support the rationale behind and importance of ensuring adequate pain management for young (and older) children. Allowing adequate time from admittance to medical intervention, when deemed medically safe, should be prioritised. As well as allowing procedures for pain management to take place, time enables HCPs to engage in the process of trust-building and permits a certain familiarity to develop.

Techniques and tools that can be used to create or facilitate the ‘space’ necessary for meaningful interaction can be developed, tested and applied, the goal being to move closer to child-centred care in practice. We suggest that research should build on the insight and experiences of HCPs who are already successfully integrating such practices into their work. At a clinical level, it may be worthwhile to designate a specific nurse to children diagnosed during their pre-school years. Building on practical experience, relevant training and the formation of professional interest groups across clinics would insure that each clinic has an expert in young children’s unique psychosocial needs.

Further, if we are to fully understand what is meaningful to young children, their own voices need to be incorporated into future research. We believe that even the voices of young children would add valuable information about their experiences and needs when they are diagnosed with diabetes. Although several researchers have developed and used innovative methods to actively include the voices of children and young people in their research [32], tools and methods aimed at preschool children are still lacking. We propose that methods used in play therapy may provide a suitable way to go forward and explore the experiences of young children.

As evident in our data, some HCPs are highly skilled at connecting with young children and putting them at ease. Perhaps the present findings would not offer these HCPs a great deal of new knowledge. However, to ensure that all children receive equal, child-centred and empathic care, this sometimes intuitive knowledge needs to be generated, studied and systematised.

Trust is likely only one of several factors that are meaningful for young children’s experience of being diagnosed with type 1 diabetes. Other needs, and how to meet them, should be considered in future research. Accordingly, the present study is a first step towards greater understanding that can inform future guidelines on the psychosocial care of young children.

### 4.2. Strengths and Limitations

The current study was open and explorative in nature. It was not our intention to examine trust per se. Thus, concepts of trust were not operationalised in our interview guide. This is both a strength and a limitation of the present study. It is a strength that we did not predefine what we believed to be meaningful to young children. We allowed our data to lead our understanding before applying a framework suitable to our findings. On the other hand, had we created our interview guide based on our chosen framework, we might have gained a better understanding of some aspects of trust, for example, whether a child-friendly environment facilitates a form of generalised trust. Self-selection of HCPs with an interest in young children and psychosocial aspects of diabetes care may have been a source of bias in the present study. In regard to the study aim, this kind of self-selection is positive. However, it may not adequately reflect general views on the importance of psychosocial care in diabetes treatment.

We wanted to try to understand the needs and experiences of young children, but without directly including their voices. Mollering [33] argue that, in a trust dyad, both members of the dyad should be interviewed, as this would enable reflection on the idiosyncrasy of trust. While not necessarily a limitation, it is important to note that the present study represents only one part of a larger picture. ‘Interviewing’ young children will require the use of some novel techniques and research approaches that have yet to be developed and tested. Further, an observation study may not be a suitable approach either, as observable outcomes of the trust process (i.e., cooperation) may not be a result of positive expectation, and similarly, unwillingness to cooperate may not be a result of negative expectation [20]. One main strength of the present study was the broad inclusion and geographical representation of all regions of Denmark. Further, the use of a theoretical framework enabled us to anchor our empirical findings to existing theoretical knowledge, offering support to both our findings and our chosen framework.

## Data Availability

Data sharing is not applicable to this article. The raw Interview data cannot be adequately anonymised in order to protect the identities of our participants.
